# A new species of *Saracha* (Solanaceae) from the Central Andes of Peru

**DOI:** 10.3897/phytokeys.85.12607

**Published:** 2017-08-09

**Authors:** Robin Fernandez-Hilario, Stacey D. Smith

**Affiliations:** 1 Herbario Forestal MOL, Facultad de Ciencias Forestales, Universidad Nacional Agraria La Molina, Av. La Molina s/n, La Molina, Lima, Perú; 2 Department of Ecology and Evolutionary Biology, University of Colorado Boulder, 1800 Colorado Ave, Boulder, CO 80309, USA

**Keywords:** *Saracha*, Solanaceae, Peru, Andes, relict forests, *Saracha*, Solanaceae, Perú, Andes, bosques relictos

## Abstract

*Saracha andina* Rob. Fernandez, I. Revilla & E. Pariente, **sp. nov.** (Solanaceae), a new species endemic to the central Andes of Peru, is described here. The new species differs from other species of *Saracha* Ruiz & Pav. by the combination of small and coriaceous leaves and clearly tubular flowers. A summary of the taxonomic history of the genus *Saracha*, an identification key for its species and a phylogenetic analysis of this genus and related genera are provided.

## Abstract (fr)

*Saracha andina* Rob. Fernandez, I. Revilla & E. Pariente, **sp. nov.** (Solanaceae), una nueva especie endémica de los Andes centrales del Perú se describe aquí. La nueva especie se distingue de las demás especies de *Saracha* Ruiz & Pav. por poseer hojas coriáceas pequeñas y flores claramente tubulares. Se proporciona un resumen de la historia taxonómica del género *Saracha*, una clave de identificación revisada para sus especies y un análisis filogenético del género y géneros afines.

## Introduction

The genus *Saracha* Ruiz & Pav. (Solanaceae) comprises two species of sometimes armed shrubs and small trees, with subcoriaceous to coriaceous leaves, pendant campanulate to tubular flowers, and fruits that blacken when mature. The species are distributed from Venezuela to Bolivia from 2200 to 4500 m.a.s.l. (Alvarez 1996, Smith and Baum 2006). Some authors have postulated that the flowers of *Saracha* are entomophilous (Cocucci 1999, Knapp 2010), but field observations indicate that at least one species, *S. quitensis* (Hook.) Miers, is pollinated by hummingbirds (Alvarez 1996, Tinoco et al. 2009).

Despite its small size, *Saracha* presents a complex taxonomic history. In 1794, Ruiz and Pavón established the genus *Saracha* in their *Florae Peruvianae et Chilensis Prodromus*, without describing any species or designating any type specimen. They presented only the generic description and an illustration. However, both match *Saracha punctata* Ruiz & Pav., which was described in the second volume of the *Flora Peruviana et Chilensis* (Ruiz and Pavón 1799). Therefore, according to Art. 40.3 of the International Code of Nomenclature (McNeill et al. 2012), *Saracha punctata* should be considered as the type species of the genus, as previously indicated by other authors (Gentry 1974, Miers 1853, Morton 1938). In the second volume, Ruiz and Pavón (1799) also described four new species, *Saracha biflora* Ruiz & Pav., *Saracha contorta* Ruiz & Pav., *Saracha dentata* Ruiz & Pav. and *Saracha procumbens* (Cav.) Ruiz & Pav., the latter four are now unanimously considered within the genus *Jaltomata* Schltdl. (Benítez 1976, D´Arcy et al. 1993, Gentry 1973, Mione and Yacher 2005, Mione et al. 2001, Mione et al. 2016).

Years later, Miers (1848) identified two groups within *Saracha*, one comprising shrub and tree species with campanulate flowers and the other comprising herbaceous species with rotate flowers. In 1848, he created a new genus (*Poecilochroma* Miers) for the first group, using *Saracha punctata* as the type. He described five new species of *Poecilochroma*, and transferred *Lycium quitense* Hook. to *Poecilochroma quitensis* (Hook.) Miers. For the second group, Miers (1849) created an amended description of *Saracha*, together with the description of ten new species and a list of previously published species.

Due to the type chosen for *Poecilochroma*, Miers had created a superfluous genus which therefore had to be rejected (Art. 52.1 of the International Code of Nomenclature; McNeill et al. 2012). In subsequent work, Miers (1853) identified his error and transferred of all recognized species in *Poecilochroma* to *Saracha*. Nevertheless, the problematic circumscriptions of the genera *Saracha* and *Poecilochroma* sensu Miers (1848, 1849) were widely used in subsequent studies (Dunal 1852, Benítez 1974, Macbride 1962, Miers 1849–1857, Walpers 1852–1853, Wettstein 1895), although these circumscriptions were disputed by some authors (Macbride 1930, 1962, Morton 1938).

The nomenclatural confusion was clarified by Gentry (1973), who restored the genus *Jaltomata*, corresponding to *Saracha* sensu Miers (1849). Further, Gentry (1974) discussed the typification of *Saracha* and reduced *Poecilochroma* to a synonym. These new re-circumscriptions of the genera *Saracha* and *Jaltomata* have been accepted and continue to be used in treatments of Solanaceae (Alvarez 1996, D´Arcy 1979, Hunziker 2001, Mione et al. 1993). Currently, the names registered in *Saracha*, most of which were published by Miers (1849, 1853) and Bitter (1913, 1921, 1922, 1924a, 1924b), have become synonyms of the two accepted species of *Saracha* or transferred to *Jaltomata* (Alvarez 1996, Mione et al. 1993). The most recent treatment of *Saracha* (Alvarez 1996) recognized only two species, *S. punctata* and *S. quitensis*, although the former is divided among three subspecies.

Recent phylogenetic studies have clarified the evolutionary history of *Saracha*. The genus falls within the fleshy-fruited subfamily Solanoideae, but is distantly related to *Jaltomata* (Olmstead et al. 1999, 2008). Indeed, the closest relatives to *Saracha* belong to the genera *Acnistus* Schott, *Dunalia* Kunth, *Eriolarynx* (Hunz.) Hunz., *Iochroma* Benth. and *Vassobia* Rusby. Together, these six genera comprise the subtribe Iochrominae (Miers) Hunz., which along with the subtribes Physalinae (Miers) Hunz. and Withaninae Bohs & Olmstead form the tribe Physaleae sensu Olmstead et al. (1999, 2008). *Saracha* species do not form a monophyletic group due to the nested placement of *Dunalia solanacea* Kunth, a species which differs dramatically in form from *Saracha* (Smith and Baum 2006, Cueva et al. 2015). Nonetheless, like *Saracha*, *D. solanacea* is restricted to the Andes and produces black fruits (unlike the remaining species of Iochrominae).

During botanical collections carried out in the Department of Ayacucho as part of the “Inventario Nacional Forestal-Ecozona Sierra” in 2015, individuals were collected with clear affinities to the genus *Saracha*. After molecular phylogenetic analysis and the review of additional material across Peru, these individuals have been recognized as a distinct undescribed species. In this article, we provide a complete description of this new species, along with ecological information and a revised identification key for the genus.

## Methods

The description was made through examination of herbarium specimens deposited in COLO, F, HSP, MO, MOL and USM (acronyms according to the Index Herbariorum, http://sweetgum.nybg.org/science/ih/), and notes taken during the study of individuals in the field. Conservation status was assigned using IUCN criteria (2012), combining field information, bibliographic data on habitat and geographic distribution based on herbarium specimens.

For molecular phylogenetic analysis, genomic DNA was extracted from silica-dried plant material (*S. Smith & R. Fernandez 594*) using the CTAB method (Doyle and Doyle 1987). We amplified and sequenced three gene regions: *LEAFY* intron II, exons 2 through 9 of the granule-bound starch synthase gene (*waxy*), and the internal transcribed spacer (ITS), following protocols described in Smith and Baum (2006). The sequences were edited and aligned to Iochrominae sequences from previous studies (e.g., Cueva et al. 2015) using MacClade 4.0 (Maddison and Maddison 2000). The Genbank numbers for *Saracha andina* sequences are KY172040 (*LFY*), KY172039 (*waxy*) and KY172041 (ITS). The phylogenetic placement of *S. andina* was inferred using maximum-likelihood analysis of the combined dataset in raxML 7.0.4 (Stamatakis 2006). We carried out model selection with likelihood ratio test in PAUP 4.0b10 (Swofford 2002) and compared the following models: JC, K2P, HKY, GTR and GTR+ Γ. We conducted a partitioned likelihood search in raxML using the best model (GTR+ Γ) and completed 100 bootstrap replicates to estimate support.

## Taxonomic treatment

### Saracha andina

Taxon classificationPlantaeSolanalesSolanaceae

Rob.Fernandez, I.Revilla & E.Pariente sp. nov.

urn:lsid:ipni.org:names:60474974-2

Figures 1 , 2

*Saracha andina affine S. punctata Ruiz & Pav*., *sed foliis coriaceus parvus, corolla tubularis et bacca ovoideus differt.*

#### Type.

**PERÚ. Ayacucho**: Prov. Lucanas, Dist. Ocaña, Centro Poblado San José de Tomate [CP Pachaca] – Sector Palca, 14°18'12.9"S, 74°45'33.11"W, 3700 m, 26 Jun 2015 (fl, fr), *E. Pariente, R. Fernandez & L. Ríos 110* (holotype MOL; isotypes MOL, USM, HSP).

#### Description.

Shrub to 2.5 m tall, widely branched from the base; younger stems, petioles and flowers pubescent with unbranched trichomes; older stems cylindrical, to 5 cm in diameter, finely striated, ash-colored; younger stems circular in cross section, 3–4.5 mm in diameter, dark, densely pubescent; internode 3–8 mm long; spines 0.9–1.5 cm long, 0.5–ca. 1 mm in diameter at the base. Leaves simple, alternate and spirally arranged, rarely geminate; petiole 2–3 (–5) mm long, planoconvex and slightly grooved, light green, moderately pubescent, but more densely so in the basal part; leaf blades (1.2–) 1.6–2.3 (–2.7) cm long, 0.6–1.4 cm wide, coriaceous, shiny, oblong to broadly elliptic, sometimes oblong-obovate, the apex obtuse, the base acute-attenuate, the margin entire and slightly revolute when dry, the adaxial surface dark green and glabrous, the abaxial surface light green with dispersed unbranched trichomes on the midrib, leaf blades concolorous when dry, the venation brochidodromus, inconspicuous, with (4–) 5–6 secondary veins. Inflorescences terminal or axillary, fascicled, with 1–2 flowers; buds ellipsoid, green with purple spots, densely pubescent. Flowers pendulous, hermaphroditic, actinomorphic; pedicels moderately pubescent, 23–27 mm long, 1–1.5 mm in diameter, green to dark purple; calyx narrowly campanulate, green to dark purple, 8.5–9.5 mm long, 4–5 mm wide, the outer surface moderately pubescent, the inner surface glabrous to minutely puberulent, the lobes 5, acute, 2–2.5 mm long, 3–3.5 mm wide, tomentose at the apex; corolla tubular, yellow at anthesis, sometimes tinged blue or purple, 25–35 mm long, 8–10 mm in diameter, the base slightly narrowed, 4–6 mm in diameter, the inner surface pubescent at the base, the outer surface densely pubescent with uniformly dispersed unbranched trichomes, the lobes 5, acute, 2–2.4 mm long, 4–6 mm wide; stamens 5, equal, filaments, white, flattened, adnate to the base of the corolla, 25–28 mm long, densely pubescent at the base, becoming glabrous at the apex; anthers oblong, 4.5–5.5 mm long, 2–3 mm wide, basifixed, with longitudinal dehiscence, the connective 4–4.5 mm long; ovary conical and glabrous, 3–3.5 mm long, 2–2.5 mm in diameter at the base; style glabrous, 17.5–18 mm long, ca. 0.5 mm wide; stigma clavate, 0.5 mm long, ca. 0.7 mm wide. Fruit a berry, ovoid and apiculate, black at maturity, 10–13 mm long, 7–8 mm in diameter, the tip 1–1.5 mm long; fruiting calyx slightly accrescent, 5–7 mm long; fruiting pedicels puberulent to moderately pubescent. Seeds not seen.

#### Distribution and habitat.

*Saracha andina* is a shrub endemic to the scrub and relict forests in the central Andes of Peru (Depts. Ayacucho, Huancavelica and Lima) at over 3500 to 4000 m in elevation (Fig. 2). *Saracha andina* grows in stony areas, on slight to moderate slopes, and near creeks. Populations of this species in the Ayacucho region have been recorded to occur in relict forest with a maximum height from 4 to 5 meters dominated by *Polylepis microphylla* (Wedd.) Bitter and accompanied by *Escallonia myrtilloides* L.f., sharing the understory with *Berberis lutea* Ruiz & Pav. and *Hesperomeles obtusifolia* (Pers.) Lindl.

#### Ecology.

Flowering and fruiting from June to September. Characteristics of the flower suggest pollination by hummingbirds (Faegri and van der Pijil 1979). In the forest where *S. andina* was collected, we observed hummingbirds such as *Metallura phoebe* and *Oreotrochilus estella*, common species in relict forests of “Queñuales” (Servat at al. 2002). These birds may be pollinators of this new species.

#### Common name and uses.

In Pachaca (Dept. Ayacucho) it is commonly known as “checc-ches” in where the native people mention that strong and straight branches had been used for yarning wool (pers. comm.).

#### Conservation status.

According to the IUCN Red List Categories (IUCN 2012), *S. andina* is classified as Endangered [EN (B1biii)]. The extent of occupancy is estimated to be less than 1,000 sqkm. Furthermore, no population of *S. andina* currently grows in any protected area and the relict forests where it lives have been reduced as result of increasing anthropogenic pressure. In this context, *S. andina* populations are highly susceptible to processes of fragmentation and degradation in short term.

#### Additional specimens examined.

**PERÚ. Ayacucho, Prov. Lucanas, Dist. Ocaña**: Centro Poblado de Pachaca, Sector Palca, 14°18'12.9"S, 74°45'33.11"W, 3700 m, 26 Jun 2015 (fl, fr), *R. Fernandez et al. 973* (HSP, MOL); Carretera Palpa-Laramate-Pachaca, ca. 3 km past Pachaca toward el puno, 14°18'12.06"S, 74°45'33.08"W, 3750 m, 09 Jul 2016 (fl), *S. Smith & R. Fernandez 594* (COLO, F, MO, USM); **Huancavelica, Prov. Huaytará, Dist. Huaytará**: Ruinas de Incahuasi, 13°34'25.77"S, 75°15'14.33"W, 3798 m, 15 Aug 2014 (fl, fr), *P. Gonzáles et al. 3385* (USM); Carretera Los Libertadores, km. 130, pasando el puente Yuraccasa, 13°34'53.07"S, 75°16'42.96"W, 3800–3850 m, 26 Jun 2001 (fl), *J. Roque & C. Arana 3309* (USM); Puente Mollepallana on road Pisco-Ayacucho, 3900–4000 m, 29 Sep 1997 (fl), *M. Weigend & H. Forther 97/604* (USM); **Lima, Prov. Canta**: Acacay, cerca a Huacoy, 29 Jul 1960 (fl), *C. Acleto 207* (USM); Ruta Canta-Obrajillo-Huacos, catarata Ongongoy, ca. 2 km NE de Huacos, 11°23'57.41"S, 76°36'11.70"W, 3900 m, 11 Jul 2016 (fl), *S. Smith 596* (COLO, MO, MOL, USM); Lachaqui, cuesta de Yacanhuana, 3800 m, 27 Mar 1973 (fl), *G. Vilcapoma 188* (MOL, USM); Lachaqui, camino a Quinana, 3900 m, 02 Feb 1979 (fl), *G. Vilcapoma 302* (MOL, USM); Huacos, catarata de Ongongoy, 3500 m, 09 Sep 2001 (fl), *G. Vilcapoma 5564* (MOL, USM).

#### Discussion.

*Saracha andina* differs from other species of the genus in its small oblong to broadly elliptic leaves 12–27 × 6–14 mm with inconspicuous nerves and tubular flowers 33.5–35 mm long. In contrast, *S. punctata* has elliptic leaves 20–150 × 8–60 mm and widely campanulate flowers, and *S. quitensis* has shorter tubular to infundibuliform flowers 12–26 mm long (Figure 3). Among other members of the subtribe Iochrominae sensu Olmstead et al. (1999, 2008), *S. andina* is perhaps most easily confused with species of *Dunalia*, many of which have tubular flowers of similar length and the plants are spiny (see discussion in D’Arcy and Smith 1987). Nonetheless, species of *Dunalia* have a diagnostic pair of appendages on either side of the filament base (“stapet”) (Hunziker 1960, 2001), and sometimes the leaves clustered on short shoots but they lack the conspicuous coriaceous leaves of *Saracha*.

**Figure 1.**
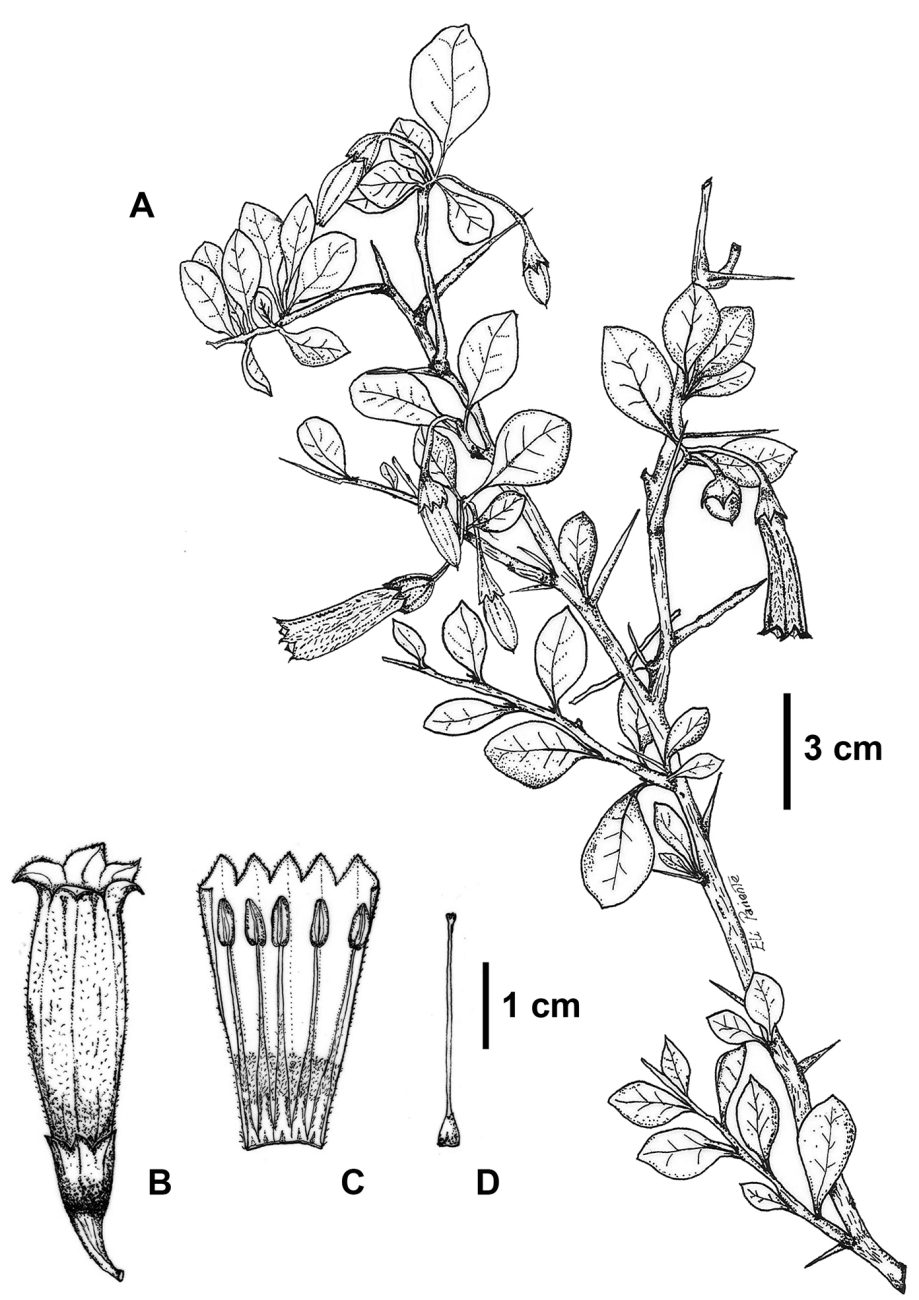
*Saracha andina*. **A** Flowering branch **B** Flower in anthesis **C** Open corolla with the stamens **D** Gynoecium. From *E. Pariente et al. 110* (MOL). Drawing by Eli Pariente.

**Figure 2.**
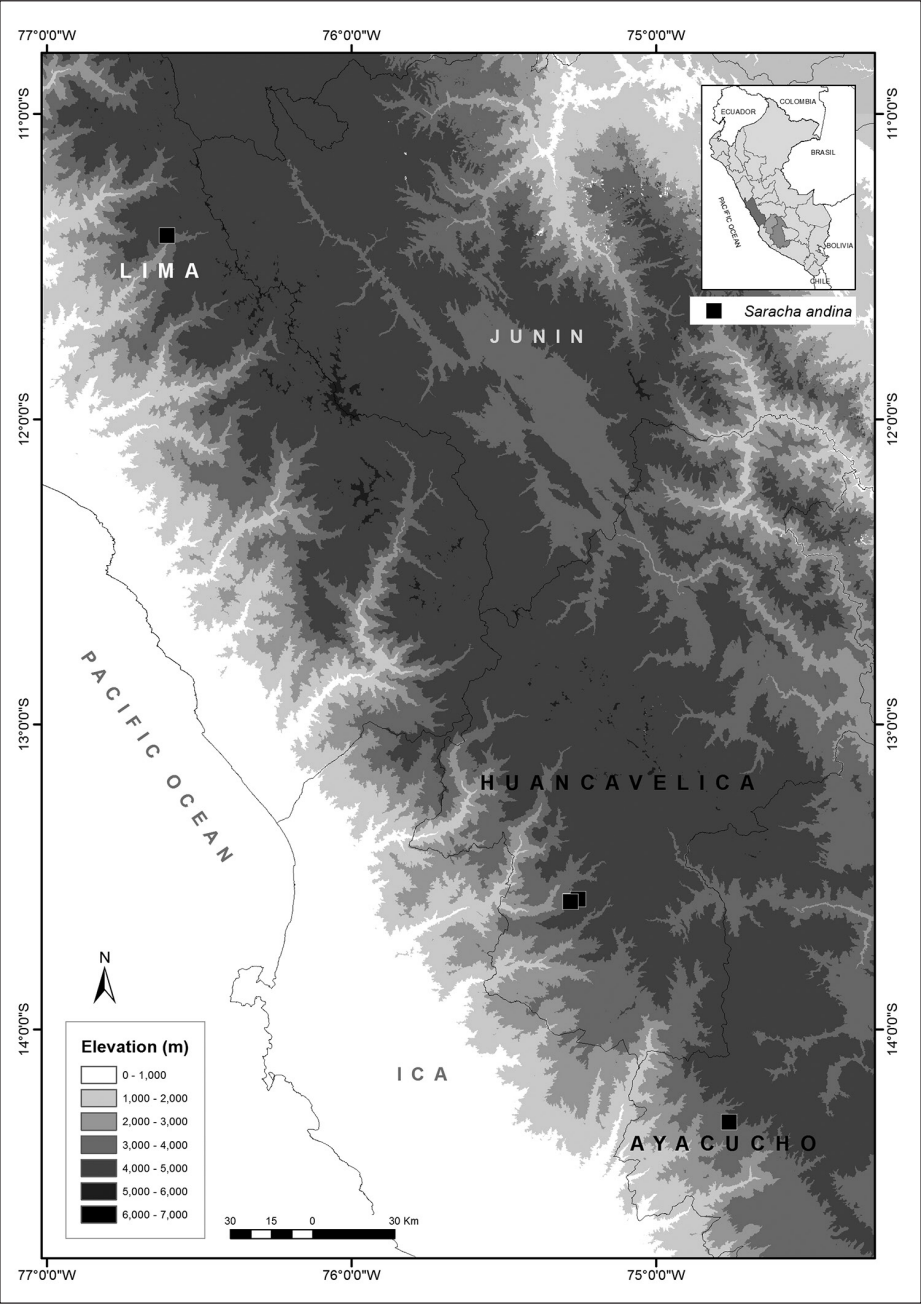
Distribution map of *Saracha andina*.

**Figure 3.**
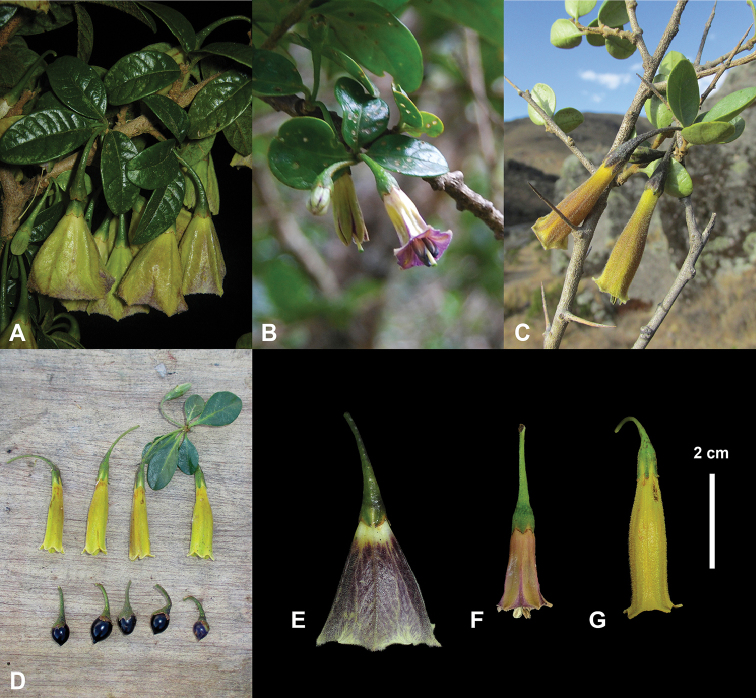
*Saracha* species. **A** *Saracha punctata* (*R. Fernandez et al. 260*; MOL) **B** *Saracha quitensis* (*S. Smith 257*; MO) **C** *Saracha andina* (*P. Gonzáles et al. 3385*; USM) **D** *Saracha andina* (*R. Fernandez et al. 973*; MOL) **E** *Saracha punctata* (*R. Fernandez 998*; MOL) **F** *Saracha quitensis* (*S. Smith 257*; MO) **G** *Saracha andina* (*R. Fernandez et al. 973*; MOL). Photos by: **A, D, E, G** Robin Fernandez; **B, F** Stacey Smith; **C** Paul Gonzáles.

**Figure 4.**
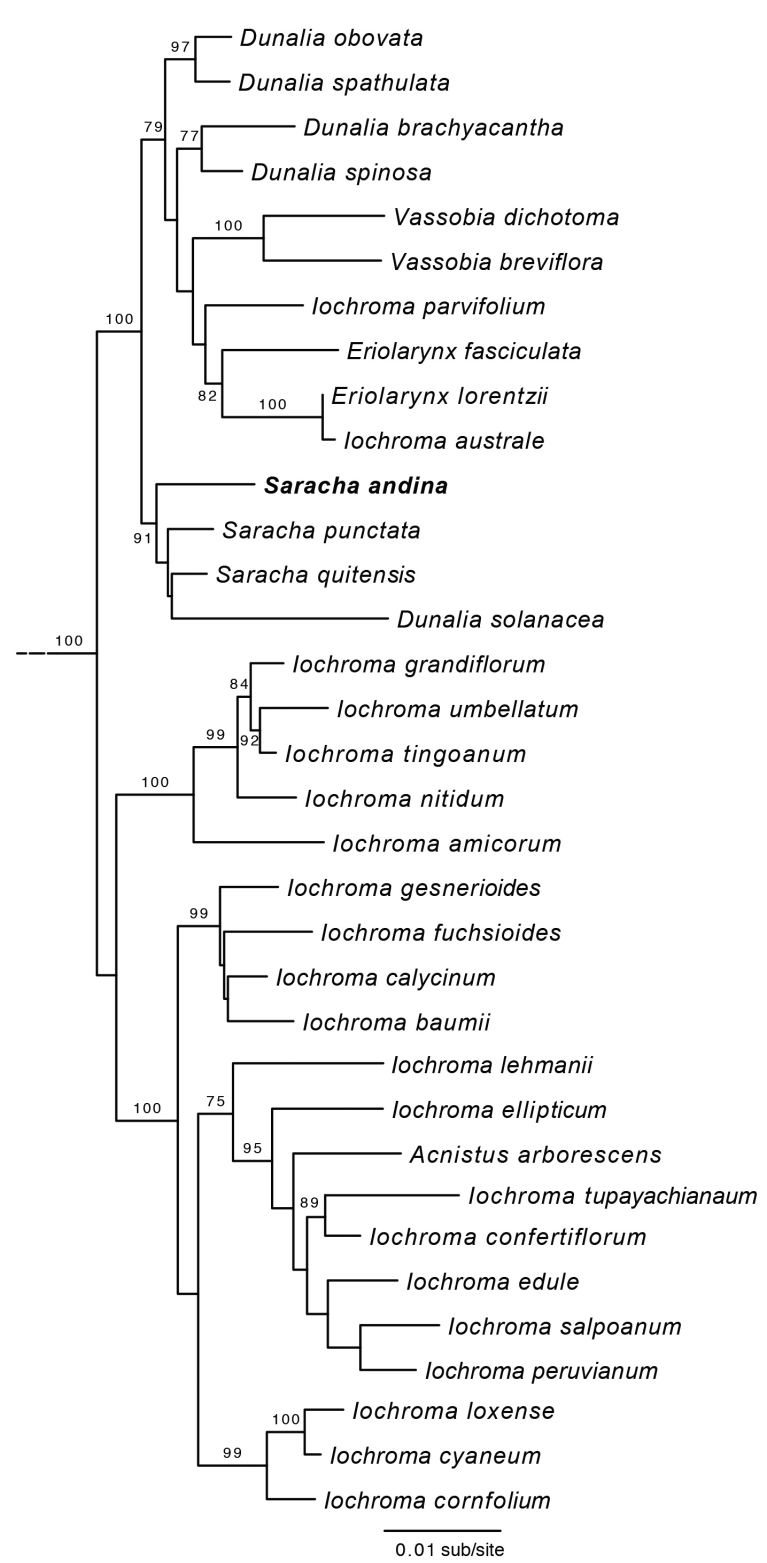
Maximum likelihood phylogeny of Iochrominae (sensu Smith & Baum, 2006) showing placement of *Saracha andina*. The outgroups (*Physalis peruviana*, *Leucophysalis grandiflora*, *Witheringia solanacea*, *Tubocapsicum anomalum*, *Cuatresia colombiana*, and *Larnax sachapapa*) have been pruned from the tree. Bootstrap support (>70%) is indicated.

### Key to the species of *Saracha*, expanded from Alvarez (1996)

**Table:** 

1	Corolla broadly campanulate, mainly yellow or yellow green, usually purple or brown spotted; leaves subcoriaceous, 20–150 mm long	***S. punctata***
–	Corolla tubular to tubular-infundibuliform, purple or yellow, sometimes with purple spots or tinges; leaves coriaceous, 10–90 mm long	**2**
2	Inflorescences with 4–10 flowers; corolla tubular-infundibuliform, 12–26 mm long; leaves 10–90 mm long; fruits globose with glabrous pedicels	***S. quitensis***
–	Inflorescences with 1–2 flowers; corolla tubular, 25–35 mm long; leaves 12–27 mm long; fruits ovoid with puberulent to pubescent pedicels	***S. andina***

The maximum likelihood phylogenetic analysis places *S. andina* together with the other members of *Saracha* with high bootstrap support (91%, Figure 4). As in previous analyses (Smith and Baum 2006, Cueva et al. 2015), *Saracha* is allied with *Dunalia*, *Eriolaryx* and *Vassobia*, all of which are distributed principally in the Andes of South America. *Dunalia solanacea* remains nested within *Saracha*, a placement supported by its black fruits, which are present in *Saracha* but absent in other *Dunalia* species (Smith and Baum 2006). Overall, these molecular results are consistent with the placement of *S. andina* in the genus *Saracha*.

## Supplementary Material

XML Treatment for Saracha andina

## References

[B1] Alvarez A (1996) Systematics of *Saracha* (Solanaceae). Master´s Thesis, University of Missouri – St. Louis, Missouri, 173 pp.

[B2] Benítez C (1974) Los géneros de las Solanaceas de Venezuela. Revista de la Facultad de Agronomía (Maracay) 7(3): 25–108.

[B3] Benítez de Rojas CE (1976) Dos nuevas combinaciones en el género *Jaltomata* Schlecht. Revista de la Facultad de Agronomía (Maracay) 9(1): 91–92.

[B4] Bitter G (1913) *Solana* nova vel minus cognita. VIII. Repertorium novarum specierum regni vegetabilis 11: 561–566. https://doi.org/10.1002/fedr.19130113406

[B5] Bitter G (1921) Zur Gliederung der Gattung *Saracha* und zur Kenntnis elniger ihrer bemerkenswerten Arten. I. Feddes Repertorium novarum specierum regni vegetabilis 17(19–30): 338–346. https://doi.org/10.1002/fedr.19210171915

[B6] Bitter G (1922) Zur Gliederung der Gattung *Saracha* und zur Kenntnis elniger ihrer bemerkenswerten Arten. II. Feddes Repertorium novarum specierum regni vegetabilis 18(4–9): 99–112. https://doi.org/10.1002/fedr.19220180406

[B7] Bitter G (1924a) Zur Gliederung der Gattung *Saracha* und zur Kenntnis elniger ihrer bemerkenswerten Arten. III. Feddes Repertorium novarum specierum regni vegetabilis 19(16–21): 265–270. https://doi.org/10.1002/fedr.19240191603

[B8] Bitter G (1924b) Zur Gliederung der Gattung *Saracha* und zur Kenntnis elniger ihrer bemerkenswerten Arten. IV. Feddes Repertorium novarum specierum regni vegetabilis 20(22–25): 362–364. https://doi.org/10.1002/fedr.19240202205

[B9] Cocucci A (1999) Evolutionary radiation in neotropical Solanaceae. In: Nee M Symon DE Lester RN Jessop JP (Eds) Solanaceae IV: Advances in Biology and Utilization. Royal Botanical Gardens, Kew, Richmond, 9–22.

[B10] Cueva M Smith S Leiva S (2015) A new and endangered species of *Iochroma* (Solanaceae) from the cloud forest of central Peru and its phylogenetic position in Iochrominae. Phytotaxa 227(2): 147–157. https://doi.org/10.11646/phytotaxa.227.2.4

[B11] D’Arcy WG (1979) The classification of the Solanaceae. In: Hawkes JG Lester RN Skelding AD (Eds) The Biology and Taxonomy of the Solanaceae. Academic Press, London, 3–48.

[B12] D’Arcy WG Smith DN (1987) *Saracha spinosa* - a new combination in Peruvian Solanaceae. Ann. Missouri Bot. Gard. 74(3): 674–675. https://doi.org/10.2307/2399334

[B13] D’Arcy WG Hunziker AT Bohs L Keel S Knapp S Mione T Nee M Rick C Spooner DM (1993) Solanaceae. In: Brako L Zarucchi J (Eds) Catalogue of the flowering plants and gymnosperms of Peru. Monogr. Syst, Bot. Missouri Bot. Gard. Vol. 45, Missouri, 1098–1137.

[B14] Doyle JJ Doyle JL (1987) A rapid DNA isolation procedure from small quantities of fresh leaf tissues. Phytochemical Bulletin 19: 11–15.

[B15] Dunal MF (1852) Solanaceae. In: De Candolle (Ed.) Prodromus Systematis Naturalis, Regni Vegetabilis, Pars XIII. Typis Crapelet, Paris, 675 pp.

[B16] Faegri K van der Pijil L (1979) The Principles of Pollination Ecology. Pergamon Press, Oxford, 244 pp.

[B17] Gentry J (1973) Restoration of the genus *Jaltomata* (Solanaceae). Phytologia 27(4): 286–288. https://doi.org/10.5962/bhl.part.13916

[B18] Gentry J (1974) The generic name *Saracha* Ruiz & Pavón (Solanaceae). Fieldiana: Botany 36(8): 69–72. https://doi.org/10.5962/bhl.title.2577

[B19] Hunziker AT (1960) Estudios sobre Solanaceae II. Sinopsis taxonómica del género *Dunalia* H. B. K. Boletín de la Academia Nacional de Ciencias 51: 211–244.

[B20] Hunziker AT (2001) Genera Solanacearum: The Genera of Solanaceae Illustrated, Arranged According to a New System. ARG Ganter Verlag KG, Königstein, 500 pp.

[B21] IUCN (2012) Categorías y Criterios de la Lista Roja de la UICN: Versión 3.1 (Segunda edición). Comisión de Supervivencia de Especies de la UICN, Gland y Cambridge, 1–34.

[B22] Knapp S (2010) On various contrivances: pollination, phylogeny and flower form in the Solanaceae. Philosophical Transactions of the Royal Society B: Biological Sciences 365: 449–460. https://doi.org/10.1098/rstb.2009.0236 10.1098/rstb.2009.0236 PMC2838263 20047871

[B23] Macbride JF (1930) Spermatophytes, mostly Peruvian-II: Peruvian Solanaceae Publication Field Museum of Natural History: 1909 – Botanical Series 8(2): 105–112. https://doi.org/10.5962/bhl.title.2340

[B24] Macbride JF (1962) Flora of Peru: Solanaceae Publication Field Museum of Natural History: 1909 – Botanical series 13(Part 5-B, Number 1): 1–267. https://doi.org/10.5962/bhl.title.2256

[B25] Maddison DR Maddison WP (2000) MacClade 4: Analysis of phylogeny and character evolution. Version 4.0. Sinauer Associates, Sunderland, Massachusetts.

[B26] McNeill J Barrie FR Buck WR Demoulin V Greuter W Hawksworth DL Herendeen PS Knapp S Marhold K Prado J Prud’homme van Reine WF Smith GF Wiersema JH Turland NJ (2012) International Code of Nomenclature for algae, fungi, and plants (Melbourne Code) adopted by the Eighteenth International Botanical Congress Melbourne, Australia, July 2011. Regnum Vegetabile 154: 1–140.

[B27] Miers J (1848) Contributions to the Botany of South America. Journal of Natural History, London: Botanical Series 3: 333–369.

[B28] Miers J (1849) Contributions to the Botany of South America. Annals & Magazine of Natural History, Ser. 2 3: 443–451.

[B29] Miers J (1853) Observations of the Solanaceae. Annals & Magazine of Natural History, Ser. 2 11: 90–105.

[B30] Miers J (1849–1857) Illustrations of South American Plants, Vol. II. H Bailliere Publisher, London, 1–150.

[B31] Mione T Anderson G Nee M (1993) *Jaltomata* I: circumscription, description, and new combinations for five South American species (Solaneae, Solanaceae). Brittonia 45(2): 138–145. https://doi.org/10.2307/2807496

[B32] Mione T Mugaburu D Connolly B (2001) Rediscovery and floral biology of *Jaltomata biflora* (Solanaceae). Economic Botany 55(1): 167–168. https://doi.org/10.2307/4256397

[B33] Mione T Leiva S Yacher L (2016) The *Jaltomata* (Solanaceae) of Department Lima, Peru. Scholars Bulletin 2(8): 476–484.

[B34] Mione T Yacher L (2005) *Jaltomata* (Solanaceae) of Costa Rica. In: Keating RC Hollowell VC Croat TB (Eds) A Festschrift for William G. D'Arcy, The legacy of a taxonomist. Monogr. Syst. Bot. Missouri Bot. Gard. Vol. 104, Missouri, 117–130.

[B35] Morton CV (1938) Notes on the genus *Saracha*. Proceedings of the Biological Society of Washington 51: 75–78.

[B36] Olmstead RG Sweere JA Spangler RE Bohs L Palmer JD (1999) Phylogeny and provisional classification of the Solanaceae based on chloroplast DNA. In: Nee M Symon DE Lester RN Jessop JP (Eds) Solanaceae IV: advances in biology and utilization. Royal Botanic Gardens, Kew, 111–137.

[B37] Olmstead RG Bohs L Migid H Santiago-Valentin E Garcia V Collier S (2008) A molecular phylogeny of the Solanaceae. Taxon 57(4): 1159–1181.

[B38] Ruiz H Pavon J (1794) Florae Peruvianae et Chilensis Prodromus. Imprenta de Sancha, Madrid, 153 pp.

[B39] Ruiz H Pavon J (1799) Flora Peruviana et Chilensis, Tomus II. Typis Gabrielis de Sancha, Madrid, 1–76.

[B40] Servat G Mendoza W Ochoa J (2002) Flora y fauna de cuatro bosques de *Polylepis* (Rosaceae) en la cordillera del Vilcanota (Cusco, Perú). Ecología Aplicada 1(1): 25–35.

[B41] Smith S Baum D (2006) Phylogenetisc of the florally diverse Andean clade Iochrominae (Solanaceae). American Journal of Botany 93(8): 1140–1153. https://doi.org/10.3732/ajb.93.8.1140 21642180 10.3732/ajb.93.8.1140

[B42] Stamatakis A (2006) RAxML-VI-HPC: Maximum likelihood-based phylogenetic analyses with thousands of taxa and mixed models. Bioinformatics 22(21): 2688–2690. https://doi.org/10.1093/bioinformatics/btl446 16928733 10.1093/bioinformatics/btl446

[B43] Swofford DL (2002) PAUP*. Phylogenetic Analysis Using Parsimony (*and Other Methods). Version 4. Sinauer Associates, Sunderland, Massachusetts.

[B44] Tinoco B Astudillo P Lattta S Graham C (2009) Distribution, ecology and conservation of an endangered Andean hummingbird: the Violet-throated Metaltail (*Metallura baroni*). Bird Conservation International 19: 63–76. https://doi.org/10.1017/S0959270908007703

[B45] Walpers G (1852–1853) Annales botanices systematicae, Tomus III. Typis JS Wassermanni, Leipzig, 1–1168.

[B46] Wettstein R von (1895) Solanaceae. In: Engler A Prantl K (Eds) Die Natürlichen Pflanzenfamilien, Teil IV, Abteilung 3b. Verlag von Wilhelm Engelmann, Leipzig, 4–38.

